# Increasing the Size-Selectivity in Laser-Based g/h Liquid Flow Synthesis of Pt and PtPd Nanoparticles for CO and NO Oxidation in Industrial Automotive Exhaust Gas Treatment Benchmarking

**DOI:** 10.3390/nano10081582

**Published:** 2020-08-12

**Authors:** S. Dittrich, S. Kohsakowski, B. Wittek, C. Hengst, B. Gökce, S. Barcikowski, S. Reichenberger

**Affiliations:** 1 Technical Chemistry I and Center for Nanointegration Duisburg-Essen (CENIDE), University of Duisburg-Essen, Universitaetsstrasse 7, D-45141 Essen, Germany; sarah.dittrich@uni-due.de (S.D.); S.Kohsakowski@zbt.de (S.K.); bilal.goekce@uni-due.de (B.G.); sven.reichenberger@uni-due.de (S.R.); 2 ZBT GmbH Zentrum für Brennstoffzellen Technik, Carl-Benz-Strasse 201, D-47057 Duisburg, Germany; 3 Umicore AG & Co. KG, Rodenbacher Chaussee 4, D-63457 Hanau, Germany; wittek.b@gmail.com (B.W.); Christoph.Hengst@eu.umicore.com (C.H.)

**Keywords:** laser ablation in liquid, nanoparticle synthesis, productivity, platinum, palladium, oxidation catalysis

## Abstract

PtPd catalysts are state-of-the-art for automotive diesel exhaust gas treatment. Although wet-chemical preparation of PtPd nanoparticles below 3 nm and kg-scale synthesis of supported PtPd/Al_2_O_3_ are already established, the partial segregation of the bimetallic nanoparticles remains an issue that adversely affects catalytic performance. As a promising alternative, laser-based catalyst preparation allows the continuous synthesis of surfactant-free, solid-solution alloy nanoparticles at the g/h-scale. However, the required productivity of the catalytically relevant size fraction <10 nm has yet to be met. In this work, by optimization of ablation and fragmentation conditions, the continuous flow synthesis of nanoparticles with a productivity of the catalytically relevant size fraction <10 nm of >1 g/h is presented via an in-process size tuning strategy. After the laser-based preparation of hectoliters of colloid and more than 2 kg of PtPd/Al_2_O_3_ wash coat, the laser-generated catalysts were benchmarked against an industry-relevant reference catalyst. The conversion of CO by laser-generated catalysts was found to be equivalent to the reference, while improved activity during NO oxidation was achieved. Finally, the present study validates that laser-generated catalysts meet the size and productivity requirements for industrial standard operating procedures. Hence, laser-based catalyst synthesis appears to be a promising alternative to chemical-based preparation of alloy nanoparticles for developing industrial catalysts, such as those needed in the treatment of exhaust gases.

## 1. Introduction

For the treatment of exhaust gases of internal combustion engines, platinum group metals (PGM) supported on γ-Al_2_O_3_ are used as standard automotive catalysts [1,2] for CO and NO oxidation. Here, it is essential to understand the activity and stability of such catalysts depending on their composition and particle size under realistic testing conditions [3]. The γ-Al_2_O_3_ support provides the advantages of a high BET surface and temperature stability under hydrothermal aging [4]. In literature, the discussion of the optimal particle size is still ongoing and partially contradictory. For instance, Boubnov et al. compared catalysts with a Pt nanoparticle (NP) size in the range of 1.2–10 nm and found an optimal particle size of approximately 2 nm for the CO conversion [5] in line with the literature [6,7]. However, results differ in the case of NO oxidation. For one, the highest activities were found at particle sizes of ~5 nm [5] and about 20 nm [8]. Graham et al. reported an increased activity for a decreased NP size and a maximal conversion rate at about 9 nm [9]. Thirdly, Xue et al. found the lowest light-off (LO) temperature and the highest maximum conversion rate to be at about 6 nm [10]. Aggravatingly, even if there would be a scientific agreement on the optimal particle size for a distinct reaction, the size of NPs does not remain constant during the lifetime of the automotive catalyst. Temperature changes during operation potentially lead to Ostwald ripening and, with further increasing temperature, coalescence [11,12,13]. Platinum nanoparticle catalysts are especially prone to these degradation processes because of the high mobility of platinum atoms under a reactive CO atmosphere [7], such that Ostwald ripening was observed in heated in situ transmission electron microscopy (TEM) studies in synthetic air [12]. Also, several research groups have reported a higher tendency of Pt NPs to form oxidized species, which reduces the adsorption of O_2_ [8,14,15,16,17,18] and CO [19] and, thus, decreases catalytic activity [5,14,20]. Even though pure Pt shows the highest catalytic activity for oxidation reactions, alloying of Pd into Pt has been shown to suppress the Pt NP growth significantly [13,17,21] by trapping mobile platinum atoms at PdO sites [7,9]. Hence, due to their better long-term stability, PtPd alloy nanoparticles are the current standard catalyst used in automotive exhaust after-treatment applications [22].

The elemental distribution (e.g., core-shell or solid solution) and alloy formation in chemical reduction methods are, amongst other factors, linked to the reduction potential differences of the mixed precursors [23,24]. Therefore, laser ablation of mixed powder or alloy targets in liquid has emerged as a promising alternative to yield alloy nanoparticles (e.g., NiMo [25], AuPt [26,27], PtCo [28], AuAg [29,30], PtPd [31]). Generally, NPs with homogeneous elemental distributions are obtained by laser ablation synthesis of colloids when fully miscible compositions are being used [32]. Laser-generated colloids are free of surfactants, i.e., organic ligands, and electrostatically stabilized by high surface charge density due to partial oxidization and adsorption of anions added in micromolar concentrations [33]. Opposed to organic ligands, the inorganic ions do not decompose into carbon, which would cover the catalytically active surface, during calcination. The barrier-less adsorption of the surfactant-free nanoparticles yields heterogeneous catalysts in quantitative efficiency, which are highly active even without heat treatment [33,34,35]. The advantages and disadvantages of different heterogeneous catalysts are described e.g., in [36]. Furthermore, the presence of lattice strains in laser-generated nanoparticles and its favorable implication in electrocatalytic reaction has recently been reported for CuZn NPs [37]. It can be assumed that lattice strains are also produced during the formation of other metal NPs due to the fast cooling conditions in the ablation plume [38].

Laser ablation in liquid has been shown to be economically favorable compared to chemical reduction synthesis when the nanoparticle productivity exceeds a material-specific value (0.5 g/h in the case of gold) [39]. Far higher productivities of 4 g/h were achieved by employing high-power 500 Watt picosecond laser ablation of platinum [40] and 1.6 g/h with a medium-power nanosecond laser system that provides the highest power-specific mass productivities of 13 mg h^−1^ W^−1^ [41]. Recently, even a power-specific productivity of more than 43 mg h^−1^ W^−1^ was found for low-priced 1 ns, 0.15 W compact lasers [42], inspiring the parallelized application of several small-scale lasers. Nevertheless, the laser pulse duration affects the nanoparticle formation pathways [38], typically causing broad size distributions in nanosecond laser synthesis [41] and bimodal nanoparticle size distribution in picosecond laser synthesis, which are typically around 5 nm and >20 nm, respectively [43]. Apart from the total nanoparticle mass productivity that is commonly discussed in the literature, the productivity of the catalytically important size fraction <10 nm is only ~30–60% of the overall productivity [41]. Hence, size quenching of laser-generated nanoparticles by in situ deposition on support particles added during laser ablation in liquid (LAL) [44,45] and post-processing steps has become well established. The in situ size quenching strategy is limited to well dispersed support materials at low concentration in order to avoid scattering of the incoming laser light or excitation of the support material potentially triggering cross-effects by changing its structure/composition. In case of the latter strategy of post-processing, the desired size fraction (<10 nm) is either extracted from the initial colloid, e.g., by continuous tubular centrifugation [41,46], or the larger fraction (>20 nm) is fragmented into smaller particles by laser fragmentation in liquid [47,48,49]. Tubular centrifugation has recently been shown to match the output flow rates of laser ablation (hundreds of mL/min), allowing it to be coupled to the ablation process downstream [46]. While laser fragmentation leads to a quantitative yield of nanoparticles <<10 nm, the throughput depends on the laser repetition rate and fluence [50]. Overall, literature shows that laser-generated NPs are not only of high interest as a catalytic material for exhaust gas treatment but also for the selective electrochemical reduction of CO_2_ [37] and the oxygen reduction reaction [28,32,51,52].

To that end, instead of only improving the overall mass-productivity, as done in previous scale-up oriented publications [40,41], this study aims to specifically scale the laser ablation process regarding the mass yield of the desired small nanoparticle fraction <10 nm. Herein, a high power ps-laser system suitable for g/h-scale nanoparticle synthesis was used to demonstrate the increased mass yield of the small particle size fraction, despite the fact that focusing the laser into the liquid decreases overall productivity. This strategy allowed for g/h-scale production and high yield of the catalytic size fraction <10 nm for the first time. To benchmark the heterogeneous catalysts gained from PtPd nanoparticles synthesized under the developed g/h conditions (regarding <10 nm size fraction), 2 kg of laser-generated PtPd/Al_2_O_3_ catalysts were prepared via adsorption of laser-generated nanoparticles to Al_2_O_3_. Honeycomb cordierite template structures were wash-coated with the catalyst, then core samples of automotive cordierite catalyst carriers were drilled out and used for catalytic runs. The performance and stability of these exhaust gas catalysts were tested in reference reactions of CO and NO oxidation within an industrial testing environment. It will be shown for the first time that the laser-generated catalysts prepared on the kg-scale show a comparable CO oxidation and even higher NO oxidation activity and stability when compared to a wet-chemically prepared reference catalyst and literature values, in an industrial test setting.

## 2. Methods and Materials

### 2.1. Pulsed Laser Ablation in Liquid

**Figure 1:**
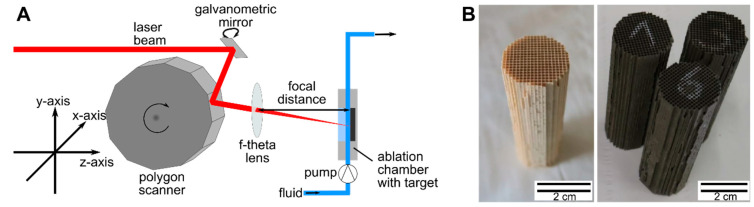
(**A**) Experimental setup of the laser-based continuous synthesis of colloidal nanoparticles (NPs); the laser beam is moved by a galvanometric mirror (slow axis), with supersonic lateral speed by a polygon scanner (fast axis) and focused by an f-theta lens on the metal target placed in the flow chamber. (**B**) Blank (left) and wash-coated (right) cordierite carriers with 3 wt.% laser-generated PtPd NP/Al_2_O_3_ catalysts.

The size selectivity of the laser ablation synthesis was optimized by focal distance and laser parameter optimization in short time experiments, aiming at the parametrization of the in-process laser fragmentation-coupled laser ablation. In the second step, longer ablation times were applied, and the particle size-optimized colloids were supported on alumina (Puralox SCFa-90, Sasol, Germany)-and wash-coated on the catalyst carrier for oxidation catalysis testing. In the size selectivity studies, the Pt NPs were fabricated by employing a 500 W ps laser (Amphos flex500, Trumpf GmbH & Co. KG, Ditzingen, Germany) in a continuously operating liquid flow setup, as described in [40] and sketched in Figure 1A.

To maximize the yield of platinum nanoparticles with a diameter below 10 nm, the distance between the target and the focusing lens (100 mm f-theta lens) was optimized between −2 mm and +2 mm, with 0 mm defined as focal distance of highest ablation rate. Here, a µm-precise translation stage was used while the applied laser pulse energy was held constant at 83.6 µJ with a 5 MHz repetition rate and 5 ps pulse duration. Laser fluence and laser intensity calculations can be found in the Supplementary Information (SI, Table S1). To maximize the ablation efficiency and nanoparticle productivity, the laser beam was scanned with a nominal scan velocity of 500 m/s in the vertical direction using a polygon scanner system. High scanning rates are required to bypass the cavitation bubble while using MHz repetition rates and to minimize scattering of subsequent laser pulses by emerging cavitation bubbles [53]. Water containing 30 µM KOH for size quenching by electrostatic interaction [54] was constantly pumped through the ablation chamber at a flow rate of 360 mL/min to maintain steady-state conditions with a constant particle concentration and size. In the size control experimental series, ablation was conducted for a total time of 2 min, while sample collection started 30 s after the ablation process was initiated. This way, the ablation rate and, hence, productivity can be determined by differential gravimetry of the target before and after ablation. To investigate the effect of in-process laser fragmentation on the productivity of nanoparticles below 10 nm normalized to the number of applied laser pulses, the focal distance was set to +1 mm in the second experimental part. Consequently, the geometric focal plane was about 1 mm in front of the target and also inside the liquid under these conditions. At a fixed laser output power of about 400 W, the applied pulse energies were varied between 263 and 42 µJ by adjusting the repetition rate of the laser between 1.5 and 10 MHz. To that end, the overall nanoparticle productivity, as well as the productivity of nanoparticles below 10 nm, were evaluated as a function of the laser fluence (pulse energy) at a constant laser power. The hydrodynamic nanoparticle diameter was characterized by an analytical disk centrifuge (CPS DC24000, CPS Instruments Inc., Oosterhout, The Netherlands). This in-process strategy, aimed to influence the particle size, was validated for laser ablation of a Pt (AGOSI Allgemeine Gold- und Silberscheideanstalt AG, Pforzheim, Germany) and PtPd alloy target (Pt_0.66_Pd_0.33_ molar composition, 3.66:1 by mass). 

### 2.2. Preparation of PtPd Catalysts and Catalytic Testing

For catalytic testing, NPs were prepared under conditions of high nanoparticle productivity, referring to the size fraction <10 nm, and laser parameters set to 400 W, 5 ps, 1.5 MHz, and 3.0 J/cm^2^. The colloid was centrifuged using a continuously operating tube centrifuge (LE GP, Carl Padberg Zentrifugenbau GmbH, Lahr/Schwarzwald, Germany) to remove the particle fraction >15 nm, in line with [41]. The large nanoparticle fraction is retained by a PTFE foil, removed from the foil, and then re-dispersed. Thus, three different catalysts were prepared from the laser-generated particles: the size-optimized monodisperse smaller NPs (later referred to as “supernatant”), the re-dispersed larger NPs (“re-dispersed”), and the non-centrifuged bimodal sample (“raw colloid”). Additionally, a “reference” catalyst, was prepared using an established wet impregnation process. The Feret diameter of more than 100 particles per sample was measured with ImageJ. The resulting data were fitted by a log-normal distribution.

Surfactant-free, laser-generated colloidal nanoparticles are easily adsorbed on a catalyst support by tuning between the diffusion-deposition and dielectrophoretic deposition regime via pH adjustment during stirring [32]. For the exhaust catalyst preparation from the laser-based Pt_0.66_Pd_0.33_ NP colloids, the γ-Al_2_O_3_ white wash coat was mixed with the respective laser-generated nanoparticle colloidal suspension with pH adjustments according to general procedures [33,35,55]. The quantitative adsorption of nanoparticles was verified as a transparent, nanoparticle-free supernatant. The mass load of Pt_0.66_Pd_0.33_ NPs on γ-alumina, which was also used for the reference catalyst preparation, was set to 2.5–3 wt.%. The mass load was confirmed by inductively coupled plasma optical emission spectrometry (ICP-OES) measurements (see Figure S1 and Table S2). The sample prepared from the supernatant obtained a slightly smaller mass-load of 2.23 wt.%. For the reference catalyst, the platinum and palladium precursor solutions were added in calculated amounts following a standardized industrial recipe. Examples of wash-coated cordierite carriers with 3 wt.% laser-generated PtPd NP/Al_2_O_3_ catalysts are shown in Figure 1B. In all cases, the wash coat load was 100 g/L. The catalysts were calcined at 550 °C for 2 h.

Catalytic tests were performed with three laser-generated NP catalysts with different PtPd NP sizes and the wet-chemically prepared reference. CO and NO oxidation were investigated in the temperature range of 75–500 °C with a temperature ramp of 15 K min^−1^. The reaction gas contained 500 ppm CO, 167 ppm H_2_, 600 ppm (as C_1_) C_3_H_6_, 200 ppm (as C_1_) C_3_H_8_, 60 ppm (as C_1_) C_7_H_8_, 140 ppm (as C_1_) C_10_H_22_, 150 ppm NO, 10% H_2_O, 13% O_2_, and 5% CO_2_ with the balance N_2_ at a space velocity of 37,500 h^−1^. To simulate the long-term performance and evaluate the catalyst stability, the catalyst-coated cordierite carriers were also tested in a second run after hydrothermal aging at 750 °C for 16 h.

## 3. Results and Discussion

### 3.1. Determination of Laser Parameter Influence on the Nanoparticle Size Fraction <10 nm

**Figure 2:**
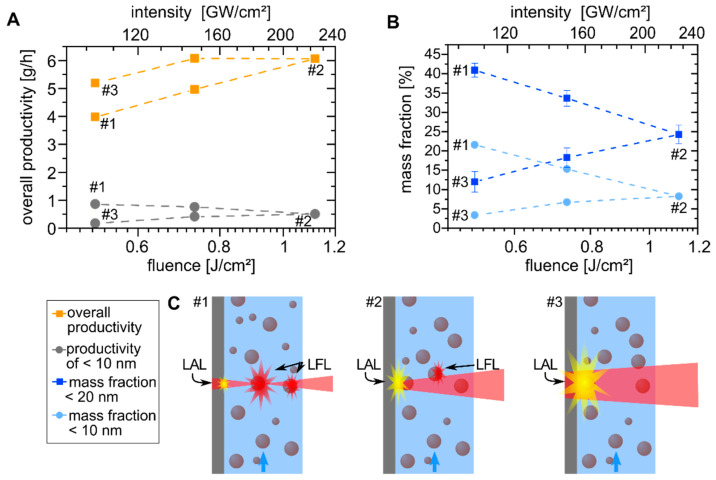
Influence of the applied laser fluence due to the change in the focal plane position on (**A**) the overall productivity (orange squares) and the productivity of the mass fraction of platinum particles smaller than 10 nm (gray circles) and (**B**) the mass fraction of NP <20 nm (blue squares) and <10 nm (light blue circles). (**C**) The sketches are related to the three different focal positions and indicate the change of laser ablation in liquid (LAL) and laser fragmentation in liquid (LFL) during focal plane shift.

The focal plane position is an essential scaling parameter for the nanoparticle productivity in laser ablation [56]. Herein, the focal position determines the nominal laser fluence applied to the target surface, where the lower fluence limit is given by the ablation threshold fluence. In turn, the upper economically feasible limit is generally constrained by the limited penetration depth of the laser light into the material or the decomposition of the liquid due to non-linear effects (e.g., filamentation) when exceeding the required threshold intensity. The latter is on the order of hundreds of GW/cm^2^ to TW/cm^2^ [57,58] for ps pulses in water and is substantially lowered if nanoparticles are present in the beam path. To that end, when changing the distance between the focusing lens and target (see sketches in Figure 2C) the highest laser fluence and the focal plane, respectively, are either located inside the liquid medium or on the target surface. In the case of the focal plane shift, a maximal intensity of about 230 GW/cm^2^ and, in the case of pulse energy variation, a maximal intensity of approximately 460 GW/cm^2^ were reached in these experiments. Although the lower thresholds of colloids compared to pure water are likely to be exceeded in our experimental conditions, the calculation of these complex concentration- and size-dependent effects are out of the scope of the present application-oriented study and will not be discussed further. Moreover, clear trends related to pulse energy and focal distance variation were observed.

When the laser spot lied inside the liquid (Figure 2C, #1), the effective laser fluence on the platinum target surface was low. Shifting the focal plane towards the target surface increased the laser fluence on the surface, reaching its maximum when the focal plane was located directly on the target surface (Figure 2C, #2). Note that a maximum laser fluence does not naturally coincide with a maximum in nanoparticle productivity, as theoretically predicted by Neuenschwander et al. [59] and experimentally proven by Streubel et al. [40], while maximum productivity correlates with an optimal laser fluence on the target surface [40,59]. Further reduction of the distance between the lens and target virtually shifted the spot behind the target, so that the fluence on the target surface decreased again (Figure 2C, #3). Interestingly, although the laser fluences of points #1 and #3 were nominally equal, the overall ablation rate was higher when the laser spot was virtually located behind the target. Waag et al. recently observed similar results for the picosecond laser ablation of nickel in water and found a decrease of the target temperature and an increased nanoparticle yield (ablation-cooling) in a similar case to that of position #3 and vice versa in the case of position #1 [60]. Complementing the discussion of Waag et al., in our study, we additionally suggest that a fragmentation process occurred in the case of #1. This is supported by the significantly increased mass fraction of particles <10 nm at #1 and consequently higher productivity of the mass fraction <10 nm, reaching more than 0.8 g/h (see Figure 2B). In turn, in the case of #3, the relative yield and absolute productivity of small particles were significantly lower. Here, the laser-induced fragmentation was strongly reduced as the fluence inside the liquid layer was the smallest for #3 (see Figure 2A,C). Post-irradiation effects of nanoparticles generated by previous pulses are likely since the average residence time of the nanoparticles inside the flow chamber is in the range of 1.5 s, which equals 7.5∙10^6^ laser pulses per volume element at the given repetition rate of 5 MHz. Hence, laser fragmentation is as expected more pronounced when the laser spot is located inside the liquid (#1). In turn, due to the fragmentation process, less laser energy reaches the target so that the overall platinum nanoparticle productivity is lower in #1 compared to #3 although similar nominal laser fluences are being applied. 

For the ablation of the Pt_0.66_Pd_0.33_ target, the same trend in the productivity of total mass as well as the catalytically relevant nanoparticle size fraction below 10 nm was found (Figure S2). Interestingly, when comparing the maximal ablation rate for Pt and Pt_0.66_Pd_0.33_, the ablation rate correlates with the material density [61] so that a similar volume-related ablation rate of about 0.27 cm³/h was found for both (see Figure 2 and Figure S2). 

**Figure 3:**
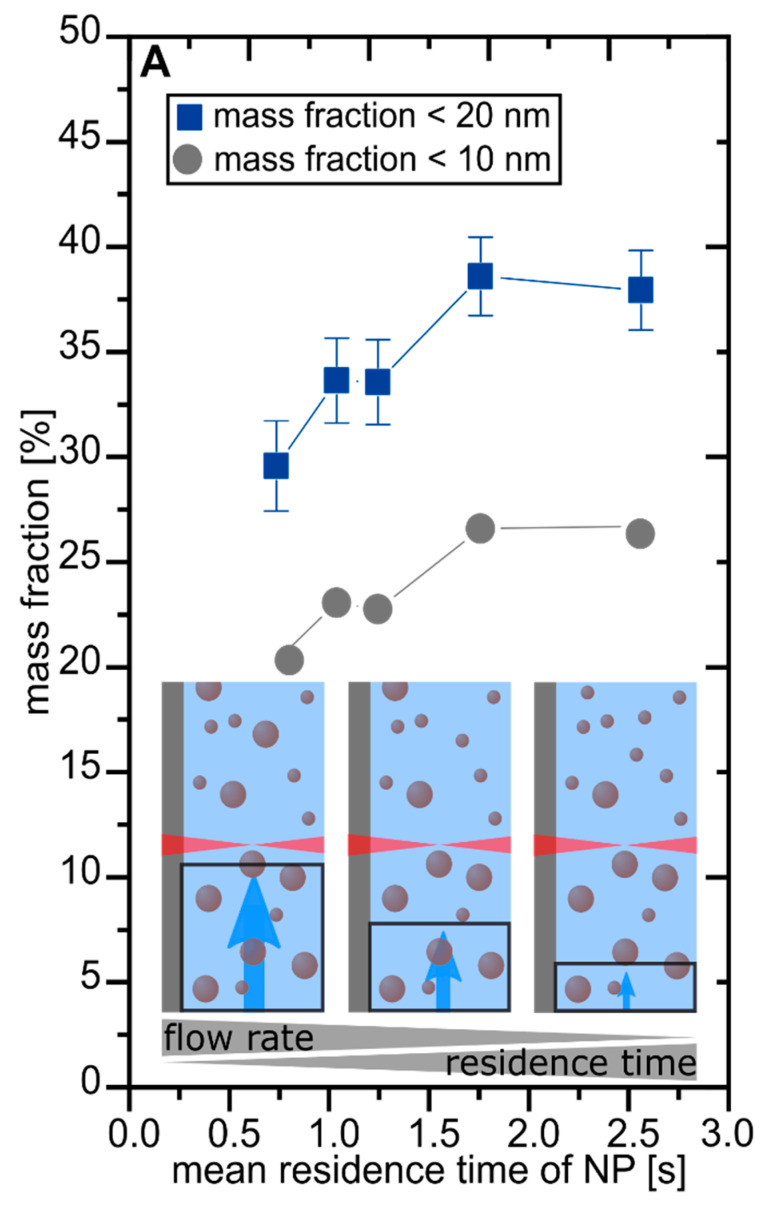
Variation of the mean residence time and the resulting mass fraction of particles <10 nm (gray circles) and <20 nm (blue squares); the sketches show how the irradiated volume decreases with increased residence time.

To verify the beneficial effect of in-process laser fragmentation on the productivity of smaller particle size fractions, the applied laser fluence was held constant, while the mean residence time of NPs in the irradiated volume was varied from 0.7–2.6 s by varying the liquid flow rate and using the Pt_0.66_Pd_0.33_ ablation target. By increasing the flow rate, a lower mean residence time of the NPs in the ablation chamber and, thus, a lower in-process laser fragmentation effect should be expected. In other words, a higher fraction of particle sizes below 10 nm is expected when the flow rate is decreased, as the mean residence time and number of post-irradiating laser pulses are increased (from 3.7 × 10^6^ to 12.8 × 10^6^ pulses per volume element). The experiments were conducted at position #1 (Figure 2C) where high fluences were applied into the liquid and laser fragmentation was expectedly pronounced. As indicated in Figure 3, the NP mass fractions (<10 nm and <20 nm) in the liquid correlate with the residence time, validating that in-process laser fragmentation triggered a significant increase in size selectivity of the laser synthesis process, although absorption cross-section and absorption efficiency of the platinum nanoparticles were low at infrared laser wavelengths [62].

**Figure 4:**
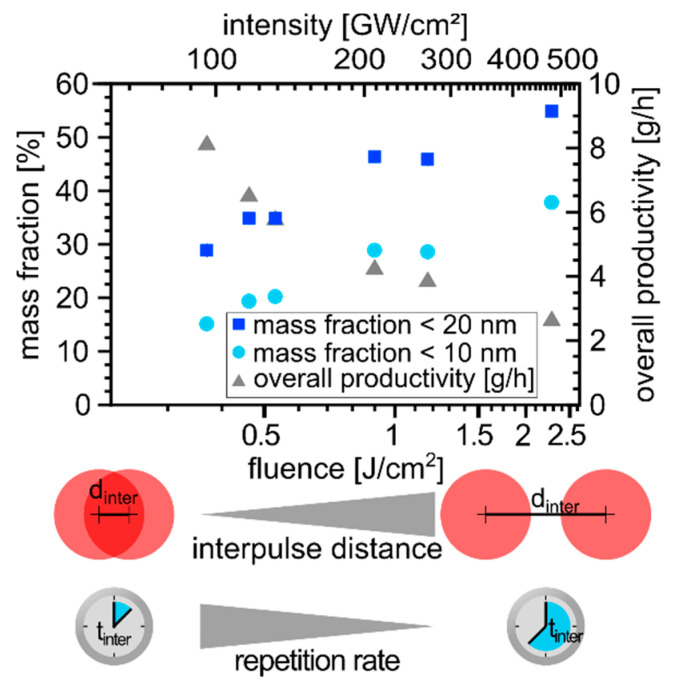
Influence of laser fluence on the mass fraction of Pt NPs <10 (light blue circles) and <20 nm (blue squares) and the overall productivity (gray triangles). The sketch below the graph indicates the change in the lateral interpulse distance d_inter_ as well as the repetition rate (inverse temporal interpulse distance t_inter_) with changing pulse energy at constant laser power.

With the previously described effect of focal distance and residence time on the size-selected nanoparticle productivity, the following question arises: how do the laser fluence (proportional to pulse energy at constant spot diameter) and the repetition rate affect the size fraction <10 nm? For the given laser system, due to $\bar{P}=E_{P}\cdot f_{R}$ , the repetition rate ( $f_{R}$ ) and laser pulse energies ( $E_{P}$ ) are anti-proportional at a constant mean laser power ( $\bar{P}$ ). Again, the working distance at position #1 was chosen as the highest productivity of the mass fraction <10 nm found at this position (see Figure 2A). The total productivity change as well as the size-selective productivity of mass fractions of <10 nm and <20 nm particles with increasing pulse energy (laser fluence) are summarized in Figure 4, respectively. Note that the overall ablation rate decreases although the ablation rate per pulse increases (Figure S3A). In contrast to the initial expectation, the productivity of NPs <10 nm (Figure S3B) remained constant at about 1.2 g/h, while the overall productivity decreased with increasing laser fluence and decreasing repetition rate (Figure 4). The latter observation agrees well with results from Streubel et al., where the optimal laser fluence for high repetition rate (10 MHz) is in the range of 0.3–0.4 J/cm^2^, similar to the fluence used at 10 MHz in this study (0.37 J/cm^2^) [40]. In turn, for the lowest repetition rate and highest fluence (1 MHz and ~2.4 J/cm^2^) in Figure 4, the fluence is about two times higher than the optimal value found by Streubel et al. for the same repetition rate (~1.2 J/cm^2^) [40]. Although the absolute production rate of the Pt NPs <10 nm did not change (compare Figure S3B), their relative mass fraction in Figure 4 significantly increased with laser fluence by a factor of more than two (compared to the same conditions at #1 in Figure 2B). 

Apart from the effect of laser fluence discussed, the decrease in the repetition rate also comes along with an increase of the lateral interpulse distance so that the pulse overlap is lowered [53]. When calculating the lateral pulse overlap from the repetition rate, focal diameter, and scan velocity, subsequent pulses obtained an overlap of about 65% for the highest repetition rate of 10 MHz at a fluence of 0.4 J/cm^2^. For the smallest repetition rate of 1.5 MHz at 2.3 J/cm^2^, no overlapping of consecutive pulses is expected. This difference in pulse overlap is sketched in Figure 4. 

Moreover, the interpulse delay (sketched in Figure 4), which is the time between two consecutive pulses, changed from 100 ns at 0.4 J/cm^2^ to 700 ns at 2.3 J/cm^2^. Due to these short time scales and the high scanning speed, the interaction of subsequent laser pulses with the cavitation bubble is unlikely. Our results indicate that a short temporal delay between two subsequent laser fragmentation pulses is beneficial to increase the yield of fragmentation. Note that fluence and interpulse delay are directly proportional so that one laser pulse fired every 700 ns with 2.3 J/cm^2^ nominally delivers the same cumulative energy to nanoparticles present in the laser’s pathway as seven pulses every 100 ns with 0.4 J/cm^2^. These observations emphasize that both the repetition rate and laser pulse energy are determinants for NP size control, particularly for MHz lasers with hundreds of nanosecond pulse delays. It is worth mentioning in this context that Plech et al. recently investigated the structural kinetics of picosecond laser fragmentation of colloidal nanoparticles in situ at time-resolved 80 ps resolution X-ray scattering. The authors reported that 54 nm gold spheres were fragmented into significantly lower particle sizes of 2–3 nm and were detected within 30 ns. Growth of the fragmented particles was observed during the first hundreds of nanoseconds, being arrested on the microsecond time scale if anions were added before laser fragmentation [63]. This indicates that the LFL process (at least of 54 nm gold) could take hundreds of nanoseconds to be completed. In other words, the interpulse delays should be long enough to allow conversion of the educt NPs into fragmented products, avoiding energy input into particles that are about to downsize from the previous pulse. Looking beyond these short time scales, barrier-less growth of the resulting ultra-small primary fragments has been observed for Pt nanoparticles after laser fragmentation, which could be suppressed (or at least slowed down) by the addition of anions [54]. Both studies [54,63] point out that particle growth quenching by anions is a perspective to further increase the yield of small particles and that the interpulse delay effects need further investigation, especially for the MHz repetition rate LFL.

Moreover, further study is also suggested for the variation of the fluence by changing the repetition rate and the pulse energy and whether high productivity of the desired mass fraction (high fluences) or high overall productivity (low fluences) is more reasonable for the application in mind. As has been systematically shown here, both can be optimized by adjusting the focal distance (located slightly in front of the target) and the laser fluence at maximum power, enabling g/h productivity. Our findings show that for the used high power (400 W) MHz-ps-laser system, the mean productivity for the catalytically important size fraction of Pt NPs <10 nm was found to be about 1.2 g/h, while the mass fraction selectivity towards small particles was freely adjustable between 5% and 40%. This size selectivity was realized by balancing between the in-process fragmentation (downsizing) and ablation (overall productivity) particle formation pathway. Further improvement of the mass fraction under a g/h-scale premise, e.g., via wavelength adaption and anion concentration variation, may be subject to follow-up studies. 

### 3.2. Examination of the Catalytic Activity of Laser-Generated PtPd Nanoparticles

**Figure 5:**
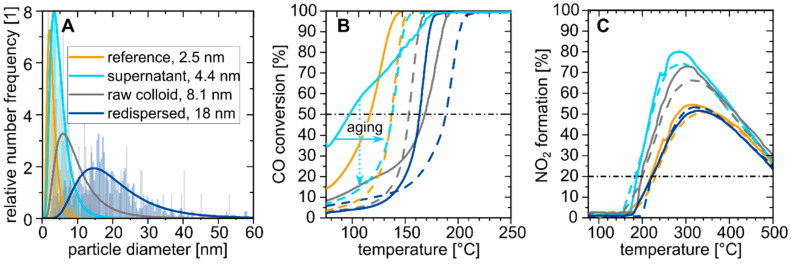
(**A**) Size distribution of the alumina-supported PtPd NPs, where the bars are the result of the transmission electron microscope (TEM) image analysis, and solid lines are the resulting log-normal fits; temperature-dependent (**B**) CO and (**C**) NO oxidation of the different PtPd catalysts. Solid lines present the first conversion run, the dashed lines are the conversion after hydrothermal aging, and the horizontal, black dotted-dashed line are the conversion at 50% and 20%, respectively. In **B**, the conversion rate change after aging is marked by two arrows, where the trend indicated by the solid arrow is valid for all curves, and the trend marked by the dotted arrow is only found for the small-sized catalysts.

To evaluate the catalytic activity of nanoparticles synthesized under these established conditions and tested in an industrial environment, laser-generated alloy Pt_0.66_Pd_0.33_ NPs were synthesized with 2.7 g/h overall and 0.9 g/h <10 nm productivity. The laser-generated nanoparticles were synthesized with the focal plane in the liquid, resulting in about 32 wt.% of <10 nm particles (see Figure S2). Through centrifugation, both fractions were separated at a 10 nm cut-off diameter to prepare catalysts containing only particles <10 nm, >10 nm, or the initial mixture of both before centrifugation (see Figures S4–S6 for more information on the NPs). In this way, NP size effects on the catalytic activity of nanoparticles stemming from the same process may be studied in addition to benchmarking with chemically prepared particles. Subsequently, the NP were supported on γ-Al_2_O_3_ with 3 wt.% load. Additionally, a reference catalyst was prepared by a wet-chemical approach. Figure 5A shows the number-weighted particle size distribution of the heterogeneous catalyst after the supporting step, as measured by TEM. The wet-chemical reference catalyst obtained a mean particle size of 2.5 nm (“reference”), whereas the laser-generated catalyst based on the raw colloid without post-processing steps achieved an average particle size of 8.1 nm (“raw colloid”). In comparison to the raw colloid, the catalysts generated with the optimized laser-generated monodisperse small particle fraction (“supernatant”, after centrifugation) and the separated big particle fraction (“re-dispersed”, after centrifugation) resulted in sizes of 4.4 and 18 nm, respectively.

The catalytic test results for the CO oxidation are shown in Figure 5B. Table 1 summarizes the temperatures at which 50% of the CO was converted during the temperature increase (temperature of 50% conversion, T_50_CO) for fresh and aged states. The fresh laser-based catalyst bearing the small particle fraction showed the best performance of all catalysts with a T_50_CO of 97 °C, compared to 117 °C for the wet-chemically synthesized reference. Due to the larger NP size, the catalyst made of the raw colloid delivered comparable activities in the first (T_50_CO value around 168 °C) and the second runs, indicating good stability. 

The T_50_CO for the reference and laser-generated small particle fraction catalysts exhibited increases by 20 K and 41 K in the run after hydrothermal aging, respectively. The disappearance of the shoulder in the first run possibly indicated absence of defects on the NP surface. For both larger-sized catalysts, the shoulder in the first catalytic run cannot be seen, which agrees with [32] that surface defects especially occur on small NPs. Note that it has recently been reported that laser-generated (palladium) nanoparticles have a higher catalytic activity than chemically prepared ones due to high defect densities caused by the kinetic-controlled nature of the synthesis process [51], at least before aging. Despite this increase in the T_50_CO and catalytic activity, the laser-based and reference catalysts revealed a similar activity after aging. Therefore, both catalysts appear to converge into similar final states. Grunwaldt et al. compared the CO/NO oxidation performance and hydrothermal aging of Pt/Al_2_O_3_ catalyst from five different preparation methods, where a superior hydrothermal resistance of the laser-generated catalyst at 500 °C was shown. However, due to small initial interparticle distances, the particle size distribution increased considerably at high temperatures [3]. 

**Figure 6:**
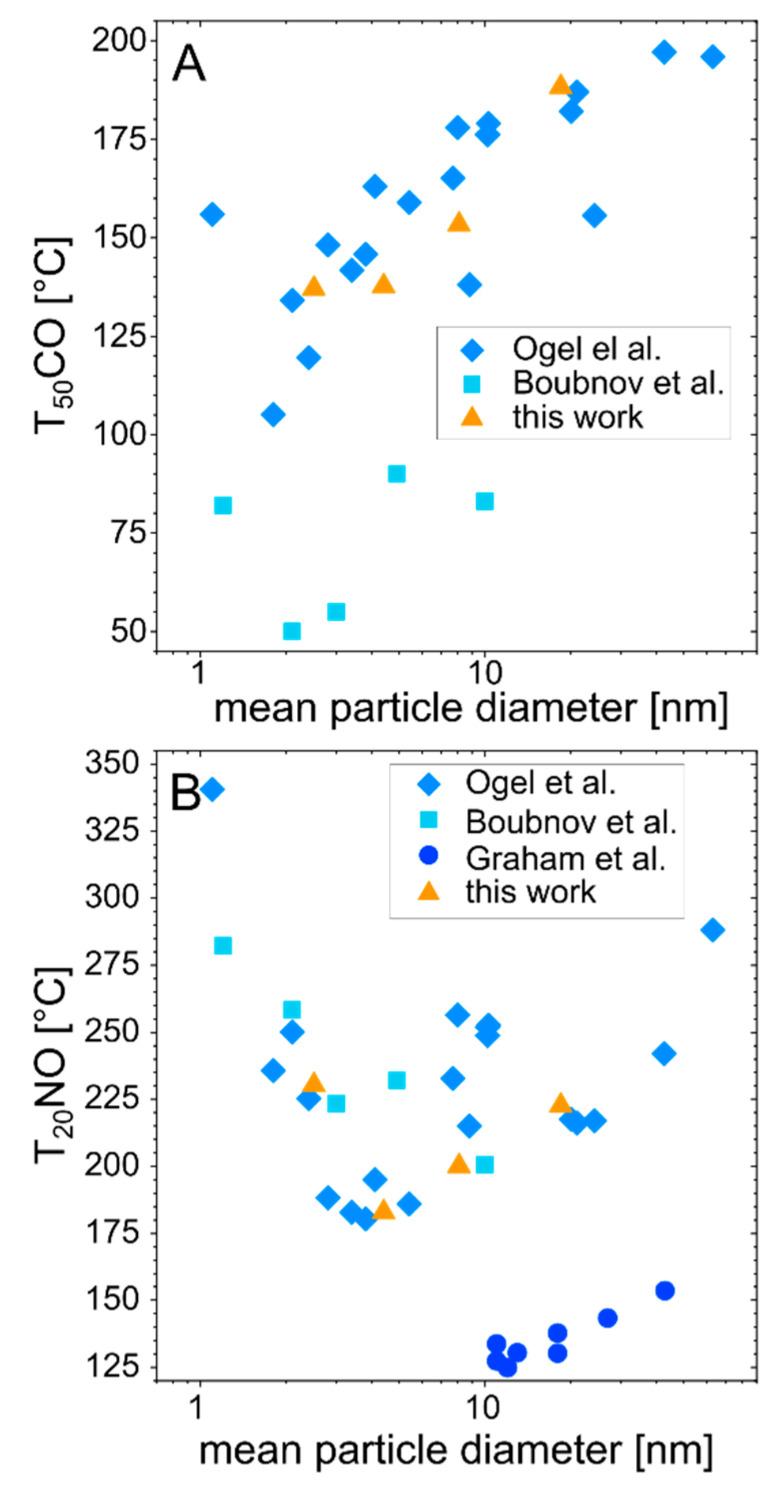
Size dependency of the (**A**) CO and (**B**) NO conversion of laser-generated catalysts; the orange triangles represent the conversion temperatures determined in this work and the blue data points are taken from the literature [3,5,9,64].

Figure 5C displays the plot of NO oxidation with maximal possible NO_2_ formation of 100%, the temperature at 20% conversion (T_20_NO), as well as the maximal conversation of NO. For the NO oxidation, the chemically prepared catalyst showed the strongest aging behavior with an increase in the T_20_NO of about 11 K. The activity of the small particle size catalyst (supernatant) on the other side was resistant to hydrothermal treatment, displaying the best overall activity of the tested catalysts with a T_20_NO of about 183 °C after aging. All size-dependent catalytic activities after aging are illustrated in Figure 6. Additionally, the catalytic results from other studies with comparable synthesis methods, reaction gas composition, and catalyst loading are described below and in Figure 6. 

Although an optimal NP size could be determined for CO and NO oxidation from Figure 6, it should be noted that the aging behavior of the different catalysts is not presented. In some of the presented studies, e.g., [9], aging has been used to alter the particle sizes. By comparing the size dependency of the NO_2_ formation for the different studies, it can be suggested that laser-generated particles perform equally well in CO and NO oxidation, similar to chemically prepared catalysts. When comparing the maximal conversion after aging, all laser-generated samples showed a higher maximal conversion of NO and yield of NO_2_. Thus, these catalysts possess a significantly higher selectivity in NO conversion, which is hypothetically linked to the particular defect-rich surface of the laser-generated PtPd nanoparticles [32,37]. The influence of different crystal structures of laser-generated Pd catalysts was experimentally demonstrated and theoretically explained by density functional theory calculations [51]. In our work, for the first time, the results in Figure 6 indicate benchmark performance of the laser-generated catalysts in both CO and NO oxidation. Yet, it should be noted that the potential contribution of the crystal structures or defect density of laser-generated compared to chemically synthesized catalysts requires further study. 

In summary, the performance of a chemically prepared reference catalyst and three different laser-generated catalysts during NO and CO oxidation were examined and compared with literature. Results reveal that small-sized catalysts are prone to aging during CO oxidation but the small-sized laser-generated catalysts show comparable conversion rates with the reference catalyst. During NO oxidation, laser-generated catalysts, although larger than the reference catalyst, display higher conversion rates, even after hydrothermal aging. The reason for the higher activity of laser-generated NPs will be investigated in a future work. 

## 4. Conclusions

The development of automotive diesel oxidation catalysts in an industrial environment requires syntheses which deliver supported alloy nanoparticle wash coats, e.g., PtPd/Al_2_O_3_, in kg-scale. Coating honeycomb cordierite carriers with the wash coats allows catalytic testing under standard exhaust gas treatment conditions. The employed nanoparticle syntheses need to operate at the g/h-scale to meet the demanded quantities of heterogeneous catalysts. For example, 1 kg of 3 wt.% PtPd supported on alumina requires about a week (30 h of 1 g/h) laser synthesis. Considering that nanoparticle sizes below 10 nm are required to enable high catalytic activity, the operation conditions that allow the g/h-scale laser synthesis of colloidal Pt nanoparticles <10 nm were investigated by tuning the size selectivity of the continuous high-power laser ablation method. It was determined that the tuning of in-process fragmentation during laser ablation is the main parameter to meet the requirements for industrial catalyst screening studies.

When shifting the focal plane of the laser beam into the liquid layer slightly above (1 mm) the ablation target, the mass yield of NP sizes <10 nm and the absolute productivity of this fraction significantly increased. This downsizing effect can be explained by an increased mean laser energy deposited in the liquid volume containing nanoparticles produced by previous laser pulses. Consequently, when the laser beam focus was located within the liquid, a higher fragmentation rate of the undesired larger Pt nanoparticle size fraction occurred, which increased the mass yield of nanoparticles below 10 nm. By increasing the laser fluence while maintaining the laser spot slightly in front of the target, the mass fraction of the small-size NP fraction increased. Moreover, reducing the volume flow rate or, in other words increasing the residence time by a factor of five, improved the mass-share of Pt nanoparticles <10 nm by more than 25%. Under these conditions, the laser fragmentation also appeared to benefit from increased temporal pulse delay, at least for the investigated MHz repetition rate scale, supported by recent literature findings on the structural kinetics of laser fragmentation. This interesting temporal effect on pulsed laser fragmentation deserves further dedicated studies. Overall, the optimized parameters allowed a continuous laser synthesis of 1.2 g/h Pt and 0.9 g/h Pt_0.66_Pd_0.33_ nanoparticles <10 nm, respectively, referring to the mass of particles below 10 nm. This equals to about 2.4 kg of 0.3 wt.% alumina-supported catalyst in an 8 h shift. After colloidal deposition of laser-generated PtPd alloy particles on alumina and coating of cordierite carriers with the former, CO/NO oxidation performance tests were conducted under conditions relevant for applications. The performance of the laser-generated catalysts was equal to that of the industrial reference and results from the literature. The laser-generated NPs even showed superior NO conversion behavior with better resistance against hydrothermal aging compared to the chemically prepared reference catalyst. The origin of the higher resistance and conversion rate may be related to the reported higher compressive strains found in laser-generated Pd nanoparticles, but this estimation requires a deeper defect-based investigation of the catalyst before and after aging as well as by analytical operando studies in the future.

## Figures and Tables

**Table 1:** Comparison of the T_50_CO and T_20_NO for the fresh and aged catalysts.

	T_50_CO (°C)		T_20_NO (°C)		Maximal NO Conversion (%)	
	Fresh	Aged	Fresh	Aged	Fresh	Aged
reference, 2.5 nm	117	137	219	230	55	51
supernatant, 4.4 nm	97	138	188	183	80	74
raw colloid, 8.1 nm	168	153	198	200	73	66
re-dispersed, 18 nm	163	188	222	223	52	54

## Supplementary Materials

The following are available online at https://www.mdpi.com/2079-4991/10/8/1582/s1, Table S1: Laser beam parameters for calculation of the laser beam fluence, Figure S1: ICP-OES measurement of the laser-generated particles supported on Al_2_O_3_, Figure S2: Influence of the change in the distance between the focal length and the ablation target position on the nanoparticle size distribution (A), the overall productivity (B), and the relative mass fraction of particle smaller than 20 and 10 nm (C) for a target made of 2:1 alloy of platinum and palladium, Figure S3: (A) Dependency of the pulse-specific productivity on the laser pulse energy and repetition rate, (B) change in the mass productivity of NPs <10 nm and <20 nm as a function of the laser pulse energy, Figure S4: Mass-weighted particle size distribution (A) and particle size fractions <10 nm and <20 nm (B) as well as the specific surface area (C) of the PtPd/Al_2_O_3_ catalysts compared in Figure 5, Figure S5: TEM histogram of the raw colloid, fitted with a log-normal distribution (blue line), moreover the cumulative number frequency (green line) and an exemplary TEM image are shown, Figure S6: TEM pictures and EDX line scans of the raw laser-generated colloid validating NP alloy formation.
