# Supplementary material for: Increasing the Size-Selectivity in Laser-Based g/h Liquid Flow Synthesis of Pt and PtPd Nanoparticles for CO and NO Oxidation in Industrial Automotive Exhaust Gas Treatment Benchmarking
Source: Nanomaterials (Basel). 2020 Aug 12;10(8):1582. doi: 10.3390/nano10081582 (PMC7466608; doi:10.3390/nano10081582)
Supplement: Supplementary file 1 [file nanomaterials-10-01582-s001.pdf]

Supplementary materials (SI) to:

Increasing the Size-Selectivity in Laser-Based g/h Liquid Flow Synthesis of Pt and PtPd Nanoparticles for CO and NO Oxidation in Industrial Automotive Exhaust Gas Treatment Benchmarking

S. Dittrich, S. Kohsakowski, B. Wittek, C. Hengst, B. Gökce, S. Barcikowski, and S. Reichenberger

Calculation of the laser fluence when varying the distance between the target and focusing lens

The angle of incidence of the laser beam on the target surface was calculated using geometric considerations and the refraction at the phase boundaries was calculated using Snell's law. The parameters used for the calculation via Eq. 1 and Eq. 2 are given by Table S1. Note that this calculation results in the nominal laser fluence (the incident fluence that reaches the target immersed in the liquid assuming plain water), whereas exact calculation of the effective fluence (the energy that is deposited in the target) would require to determine the laser attenuation in the colloid volume of the beam path reaching the target (with the attenuation depending on nanoparticle concentration, nanoparticle size distribution and eventually self-focusing effects) as well as the temperature-dependent target reflectivity at the given wavelength.

$$\Phi = \frac{2 \cdot E_P}{\pi \cdot w_0^2} \qquad \text{Eq. 1}$$

$$I = \frac{2 \cdot P_P}{\pi \cdot w_0^2} \qquad \text{Eq. 2}$$

Φ: fluence [J/cm²]

E_P: pulse energy [J]

w_0: 1/e²-beam radius [cm]

I: laser intensity [W/cm²]

P_P: pulse power [W]

Table S1**: Laser beam parameters for calculation of the laser beam fluence**

**parameter**	**value**
spot diameter on the target surface at the focal distance of maximal productivity [39]	69 µm
experimentally determined focal distance between the lens and the target surface at the point of maximal productivity	124.4 mm
width of the ablation chamber window	2.2 mm

refractive index of the ablation chamber window (N-BK7), taken from data sheets provided by SCHOTT North America, Inc.	1.5071
width of the liquid layer over the ablation target	5 mm
refractive index of water [63]	1.3265

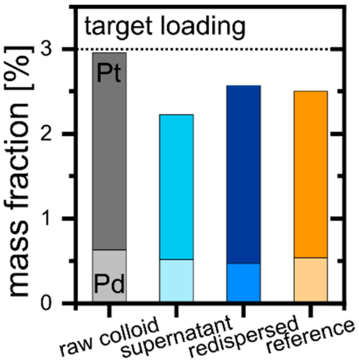

Figure S1: ICP-OES measurement of the laser-generated PtPd particles supported on Al_2O_3

Table S2: NP composition of the of the different PtPd/Al_2O_3 catalysts, obtained by ICP-OES measurements

	Pt	Pd
raw	3.69	1
supernatant	3.30	1
redispersed	4.45	1
reference	3.66	1

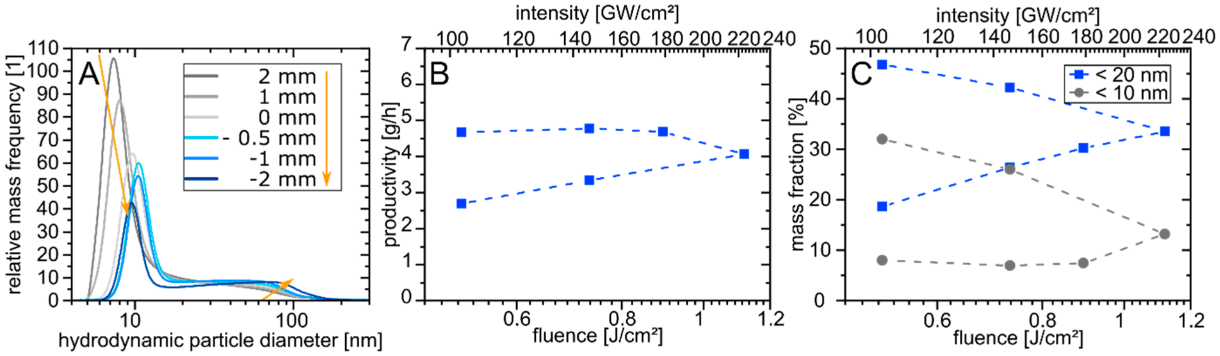

Figure S2**:** Influence of the change in the distance between the focal length and the ablation target position on the nanoparticle size distribution (**A**), the overall productivity (**B**), and the relative mass fraction of particle smaller than 20 and 10 nm (**C**) for a target made of 2:1 alloy of platinum and palladium

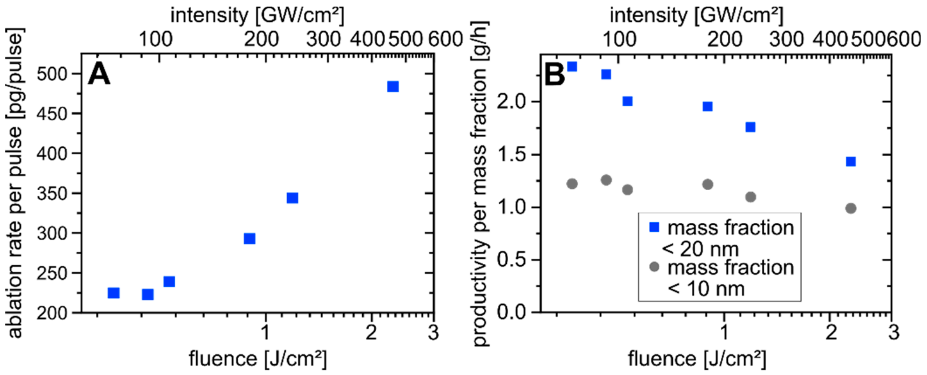

Figure S3**:** (**A**) Dependency of the pulse-specific productivity on the laser pulse energy and repetition rate, (**B**) change in the mass productivity of NPs <10 nm and <20 nm as a function of the laser pulse energy

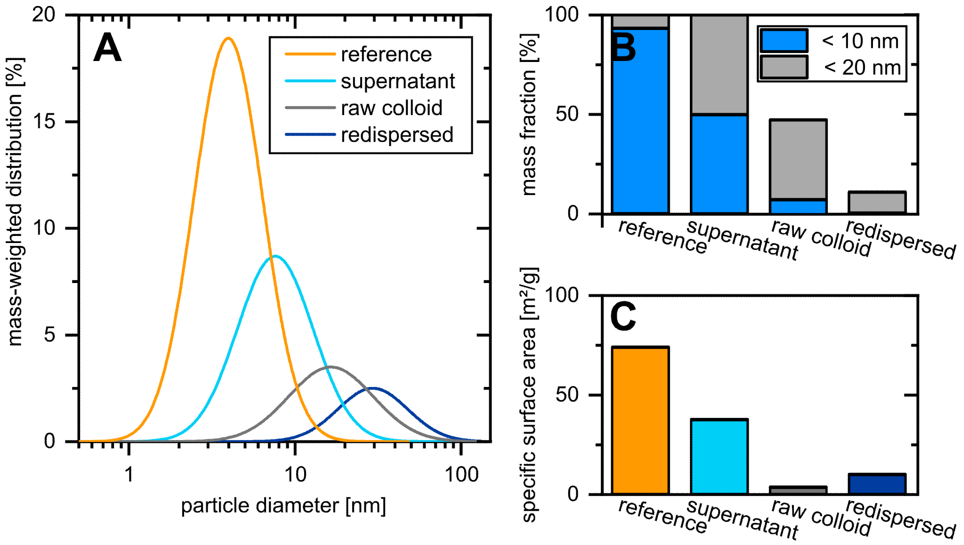

Figure S4**:** Mass-weighted particle size distribution (**A**) and particle size fractions <10 nm and <20 nm (**B**) as well as the specific surface area (**C**) of the PtPd/Al_2O_3 catalysts compared in **Error! Reference source not found.**

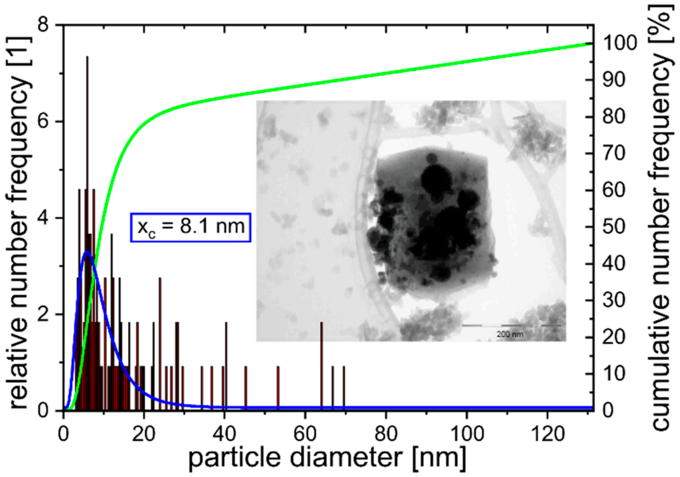

Figure S5: TEM histogram of the raw colloid (PtPd/Al_2O_3), fitted with a log-normal distribution (blue line), moreover the cumulative number frequency (green line) and an exemplary TEM image are shown

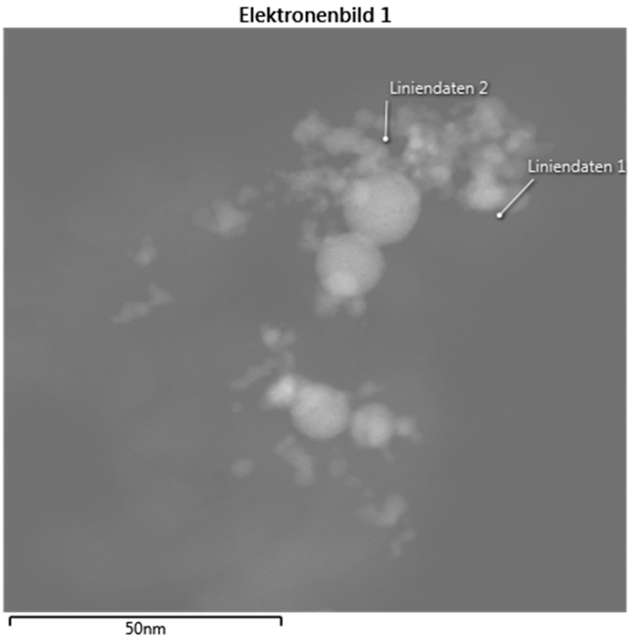

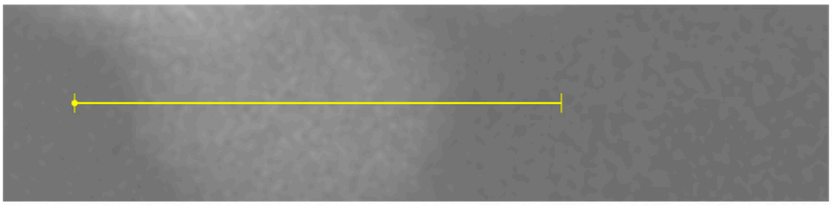

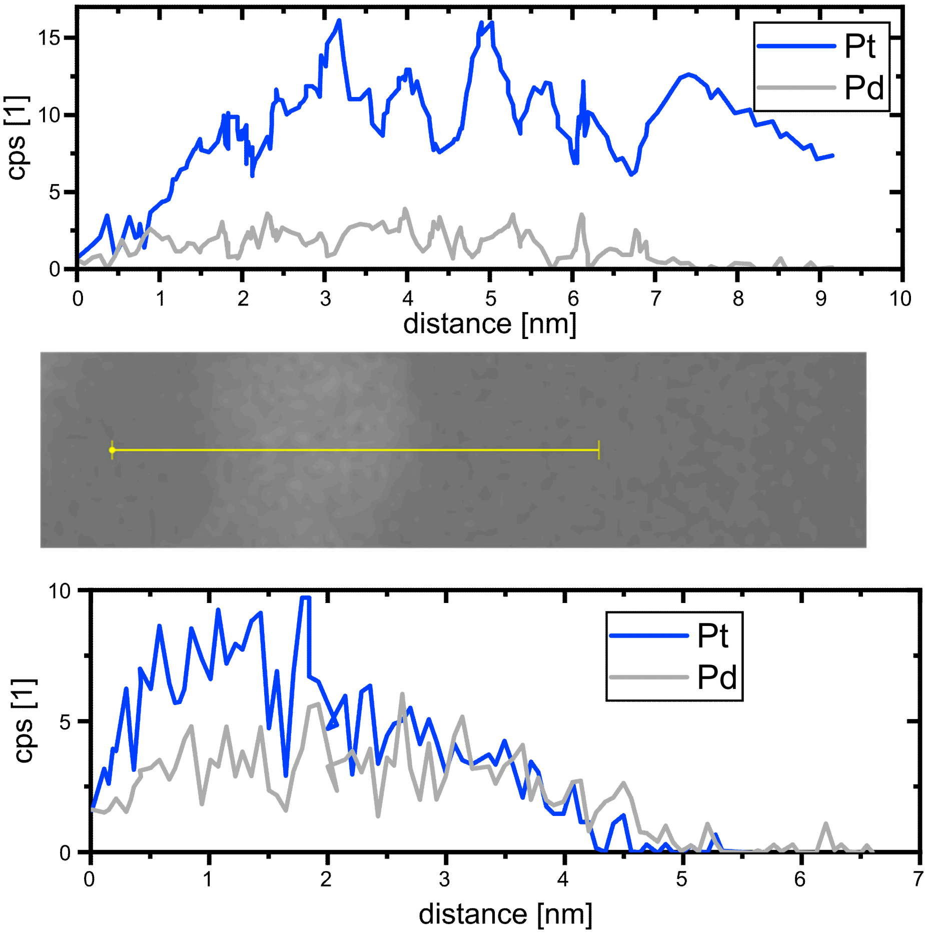

Figure S6: TEM picture and EDX line scans of the raw laser-generated colloid validating NP alloy formation
